# Baseline Antibody Titre against* Salmonella enterica* in Healthy Population of Mumbai, Maharashtra, India

**DOI:** 10.1155/2017/9042125

**Published:** 2017-08-31

**Authors:** Rucha Patki, Sunil Lilani, Dhaneshwar Lanjewar

**Affiliations:** ^1^Grant Government Medical College and Sir J. J. Group of Hospitals, Mumbai, Maharashtra, India; ^2^Shri Bhausaheb Hire Government Medical College and Hospital, Dhule, India; ^3^Department of Pathology, Grant Government Medical College and Sir J. J. Group of Hospitals, Mumbai, Maharashtra, India

## Abstract

**Objective:**

The aim of this study was to establish a baseline titre for the population of Mumbai, Maharashtra, India.

**Method:**

Four hundred healthy blood donors, attending blood donation camps, were screened using a survey questionnaire. Widal tube agglutination test was performed on the diluted sera (with 0.9% normal saline) of blood donors, with final dilution ranging from 1 : 40 to 1 : 320.

**Results:**

Out of 400 individuals providing samples, 78 (19.5%) individuals showed antibody titres ≥ 1 : 40 for at least one antigen and 322 (80.5%) showed no agglutination. The baseline antibody titres against O antigen and H antigen of* Salmonella enterica *serotype Typhi were found to be 1 : 40 and 1 : 80, respectively. Similarly, the baseline antibody titres for the H antigen of* Salmonella enterica *serotypes Paratyphi A and Paratyphi B were found to be 1 : 40 and 1 : 80, respectively.

**Conclusion:**

Thus, it was noted that the diagnostically significant cutoff of antibody titre from acute phase sample was ≥ 1 : 80 for* S. *Typhi O antigen and titre of ≥ 1 : 160 for both* S. *Typhi H antigen and* S. *Paratyphi BH antigen. Antibody titre of ≥ 1 : 80 can be considered significant for* S. *Paratyphi AH antigen.

## 1. Introduction 

The term enteric fever includes typhoid fever, caused by* Salmonella enterica* subspecies* enterica *serotype Typhi and paratyphoid fever caused by* Salmonella enterica* subspecies* enterica* serotypes Paratyphi A, Paratyphi B, and Paratyphi C [1]. Endemic in India, there are an estimated 6,345,776 cases per year of enteric fever with case fatality rate <1% [2]. The criterion standard for diagnosis of enteric fever has long been culture isolation of the organism [3]. Among these, the isolation of bacteria from blood or bone marrow is considered the gold standard. However, in developing countries, culture facilities are not easily available or accessible and serological tests such as Widal test remain the mainstay of diagnosis. The Widal test detects the presence of antibodies against antigens of* Salmonella enterica* in the blood. However, in healthy populations of the endemic areas, the antibody titre may be found to be high in the presence of cross-reacting antigens owing to other infections, a healthy carrier state, long-standing liver disease, and previous typhoid vaccination [4]. Therefore, it becomes necessary to collect sera from an acute phase and a convalescent phase, at least 10–14 days apart to demonstrate a fourfold rise in the antibody titres [5]. However, a rise in the titre of antibodies cannot always be substantiated on account of early rise in antibodies due to high endemicity, late collection of samples in the natural history of disease, or prior intake of antibiotics [6–8]. Furthermore, an anamnestic response may develop during an acute febrile illness, such as malaria, in people with past* Salmonella *infection or history of vaccination against* Salmonella* [1]. In order to use the Widal test effectively, it is required that an accurate baseline titre is obtained for the diagnosis of enteric fever, so as to interpret the antibody titre results from a single acute phase sample against the baseline Widal titre present in healthy individuals in that particular geographical area [8, 9]. Substantial differences in baseline Widal titres are present in healthy individuals of different geographical areas, depending on the endemicity of enteric fever in those areas, as determined by studies done in other states of India such as Karnataka, Uttarakhand, Gujarat, and Kerala [10–13]. The baseline antibody titre has not been established for the Mumbai region. In view of the above, this study was carried out to ascertain the baseline antibody titre, in the geographical area of Mumbai city of Maharashtra state, India, taking into consideration the fact that enteric fever is a major public health problem in Mumbai, India.

## 2. Materials and Methods 

This study was a cross-sectional type of study carried out at the Department of Microbiology, Grant Government Medical College and Sir J. J. Group of Hospitals, Mumbai, Maharashtra, India, after obtaining an approval from the Institutional Ethics Committee. Blood samples were collected after obtaining written informed consent from healthy blood donors (*N* = 400), of both genders and those aged 18 to 55 years, who attended the various blood donation camps organized by the institution during the period extending from May 2014 to September 2014.

The blood donors were screened using a survey questionnaire. Blood samples were collected from apparently healthy donors, who had neither been vaccinated with TAB (Typhoid and Paratyphoid A and B) vaccine or oral typhoid vaccine/Vi vaccine nor had suffered from enteric fever in the past. We selected only those blood donors who were permanent residents of Mumbai, India.

Individuals with any active or recent infections including hepatitis B, hepatitis C, malaria, Dengue fever, brucellosis, and HIV/AIDS and any type of fever in the last 6 months were excluded from this study.

A venous blood sample of 5 ml was collected in a plain tube and was allowed to clot at room temperature for about 30 minutes. The samples were then centrifuged at 3000 rpm (revolutions per minute) for 10 minutes in order to separate the serum from the blood. Sera were transferred to clean and dry sterile storage vials. The serum samples were refrigerated at −20°C. Stained* Salmonella *antigen test kit for tube test was provided by Star Diagnostics Pvt. Ltd., India. This test kit contained smooth suspensions of antigens “O” and “H” of* Salmonella* serotype Typhi, “H” antigen of* Salmonella* serotype Paratyphi A, and “H” antigen of* Salmonella* serotype Paratyphi B.

The test was performed after bringing sera and reagents to room temperature. Widal titre was estimated by confirmatory quantitative tube agglutination test using standard agglutination test procedure [4]. As per the manufacturer's instructions, 0.5 ml of the increasingly diluted sera, dilutions ranging from 1 : 20 to 1 : 320 in 0.9% normal saline, was tested by adding an equal amount (0.5 ml) of antigen, thereby giving final dilution ranging from 1 : 40 to 1 : 640, and the tubes were then incubated overnight at 37°C in a water bath. Saline and positive controls were added in every set of the test. The tubes were mixed well and incubated in a serological water bath maintained at 37°C for 16–20 hours and were observed for agglutination. In a positive test, O antigen showed granular agglutination and H antigen showed fluffy agglutination. The last tube showing visible agglutination with the naked eye was taken as an endpoint of the test. The titre was reported out as the reciprocal of the endpoint. Absence of agglutination suggested a negative test.

The method used for finding the baseline titre was Widal tube agglutination test. The amount of antigen suspension used was the same as the amount of diluted sera making the dilution 2-fold, and hence the readings in our study begin with titre 1 : 40, which is in accordance with the manufacturer's instructions and also the standard lab protocol followed by the Department of Microbiology of the institution where our study was conducted.

### 2.1. Statistical Analysis

Data was analyzed in an Excel sheet and the variables were summarized using frequency count and percentage.

## 3. Results 

A total of 400 apparently healthy blood donors provided serum samples for testing the baseline antibody titre against* Salmonella enterica*, using Widal tube agglutination test. Blood donors were in the age group of 18 to 55 years. Out of all participant blood donors, 352 (88%) were males. Out of the 400 individuals, 78 individuals (19.5%) were found to be positive (≥1 : 40) for at least one of the antigens. 322 individuals (80.5%) were found to be negative (<1 : 40) for all antigens (Table 1).

As seen in Table 2, among 400 individuals, a total of 37 (9.25%) individuals were found positive (≥1 : 40) for* S. *Typhi “O” antigen and 41 (10.25%) were found positive (≥1 : 40) for* S. *Typhi “H” antigen. Only 2 (0.5%) individuals were positive (≥1 : 40) for* S. *Paratyphi AH antigen. The total number of individuals positive (≥1 : 40) for* S. *Paratyphi BH antigen was 15 (3.75%).

As seen in Table 3, among the 37 (9.25%) individuals who were found to be positive against O antigen of* S. *Typhi, 20 (5%) individuals had a titre of 1 : 40, 14 (3.5%) individuals had a titre of 1 : 80, 1 (0.25%) individual had a titre of 1 : 160, and 2 (0.5%) individuals had a titre of 1 : 320. Similarly, among the total of 41 (10.25%) individuals positive against H antigen of* S. *Typhi, 11 (2.75%) individuals had a titre of 1 : 40, 25 (6.25%) individuals had a titre of 1 : 80, and 5 (1.25%) individuals had a higher titre of 1 : 160.

As seen in Table 3, out of the 2 (0.5%) individuals who tested positive against H antigen of* S. *Paratyphi A, 1 (0.25%) individual had a titre of 1 : 40 and the other individual had a titre of 1 : 80. It can be seen that very few individuals (0.5%) were positive for H antigen of* S. *Paratyphi A.

Among 15 (3.75%) individuals positive against H antigen of* S. *Paratyphi B, 4 (1%) individuals had a titre of 1 : 40, 7 (1.75%) had a titre of 1 : 80, and only 4 (1%) samples reported a higher titre of 1 : 160 (Table 3).

## 4. Discussion 

Isolation of* Salmonella* from blood or bone marrow has always been the gold standard for diagnosis of enteric fever. However, most healthcare centers in India do not have easy access to this method and also culture methods are seen as time-consuming. The Widal agglutination test has always been the preferred test for diagnosis of enteric fever in many developing countries, including India, where it is extensively practiced. Especially in endemic areas, the Widal test is still of significant diagnostic value, provided judicious interpretation of the test is made against a background of pertinent information, especially data which relate to agglutinin levels in normal individuals and in nontyphoidal fevers common in the region [8].

This study was the first ever study done in Mumbai, to the best of our knowledge, which was conducted for the estimation of baseline antibody titre in healthy individuals against serotypes Typhi and Paratyphi A and Paratyphi B of* Salmonella enterica*. Our study has shown that disparate titres of antibody, against the different serotypes of* Salmonella enterica,* exist in healthy individuals.

The results of our study established the presence of high antibody titres in healthy individuals. Out of 78 (19.5%) healthy individuals with positive titres for at least one antigen, we found that 37 (9.25%) had anti-O antibody titres of ≥ 1 : 40 and 41 (10.25%) had antibody titres of ≥ 1 : 80 against* S. *Typhi H antigen. Taking these results into consideration, the baseline antibody titres against O antigen and H antigen of* Salmonella enterica *serotype Typhi were found to be 1 : 40 and 1 : 80, respectively. Our results are in concordance with those reported in studies conducted by researchers in other states of India that the disease is endemic to, such as Karnataka and Uttarakhand, where the recorded baseline titres against O antigen and H antigen of* S. *Typhi were found to be 1 : 40 and 1 : 80, respectively, in both states [10, 11]. A similar study was done in Nepal by Pokhrel et al., which was the first study done in Nepal to determine the baseline titre and cutoff values for the diagnosis of enteric fever. It was concluded that significant titres are those higher than 1 : 80 for anti-O and higher than 1 : 160 for anti-H antigen of* Salmonella enterica* serotype Typhi, for a possible diagnosis of typhoid fever for Kathmandu Valley, Nepal [14].

In this study, the greatest anti-O and anti-H titres against* S. *Typhi were 1 : 320 and 1 : 160 in 0.5% and 1.25%, respectively. Such higher titres of 1 : 160 against O and H antigens of* S. *Typhi were seen in a study carried out in Kerala, India, by Sreenath [13]. We obtained a very low number of individuals showing antibody titres against H antigen of* S.* Paratyphi A. This study demonstrated that anti-H agglutinin titre to* Salmonella enterica *serotypes Paratyphi A and Paratyphi B in Mumbai was lower than that against* Salmonella enterica* serotype Typhi. A total of 15 individuals had anti-H titre ≥ 1 : 40 to* Salmonella enterica* serotype Paratyphi B. A very little proportion of individuals showed significant levels of anti-H titre against* Salmonella enterica* serotype Paratyphi A, indicating that it is not a usual serotype in Mumbai.

Widal agglutination test can be positive in individuals with previous immunization against* Salmonella *antigen, infection with other Enterobacteriaceae, and other diseases such as malaria and Dengue fever and also due to variability and poor standardization of commercial antigen preparation. False positive agglutination can also be seen as a cross reaction with nontyphoidal* Salmonella* [15]. A study conducted in Papua New Guinea by Clegg et al. was able to clearly demonstrate a remarkable change in the immune status of the community, in which those healthy individuals with a Widal tube O agglutination titre of 40 or more rose from 0 to 56% in the short span of five years. It was found that the factors influencing the usefulness of the Widal agglutination test in a population include the endemicity of typhoid fever, the immune response to typhoid, baseline antibody levels of the general population to* Salmonella *Typhi, and the ability to select an appropriate diagnostic cutoff titre [16]. Though its usefulness will vary with time and place, the Widal agglutination test remains a useful diagnostic test under appropriate circumstances. Hence, establishing and updating the baseline Widal titre for a particular area are an important factor in the proper interpretation of the Widal test. Thus, assessment of the Widal baseline titre for healthy individuals in a specific geographical area should be performed from time to time.

Various studies which have been carried out in different states of India have shown that the baseline titre is different in different geographical locations. Hence, the baseline titre of a specified geographical area should first be established [10–12]. Typhoid fever remains a major public health problem in developing countries like India [17]. Being inexpensive and readily available, Widal tube agglutination test will probably remain the preferred diagnostic test in many developing countries, such as ours, understanding that the baseline antibody titre is established.

## 5. Conclusions 

Widal test results from an acute phase serum sample should be interpreted based on the baseline titre prevalent in healthy individuals in that particular geographic area. In this study, the baseline antibody titres against O antigen and H antigen of* Salmonella enterica *serotype Typhi were found to be 1 : 40 and 1 : 80, respectively. Similarly, the baseline antibody titres for the H antigen of* Salmonella enterica *serotype Paratyphi A and* Salmonella enterica *serotype Paratyphi B were found to be 1 : 40 and 1 : 80, respectively. Thus, an antibody titre, from acute phase sample, of ≥ 1 : 80 for* S. *Typhi O antigen and titre of ≥ 1 : 160 for both* S. *Typhi H and* S. *Paratyphi B H antigens and antibody titre of ≥ 1 : 80 for H antigen of* S. *Paratyphi A can be considered as diagnostically significant.

## Supplementary Material

The participant blood donors were surveyed using a questionnaire. Only those who had never been vaccinated with TAB or oral typhoid vaccine and who had not suffered from enteric fever in the past, were selected for the study. Participating blood donors were informed and explained about the study and consent was obtained.

## Figures and Tables

**Table 1:** Widal test results.

Widal status	Number of individuals (*N* = 400)
AB titre ≥ 1 : 40	78 (19.50%)
AB titre < 1 : 40	322 (80.50%)

*N*: total number of participant blood donors; AB: antibody.

**Table 2:** Distribution of positive samples (antibody titre ≥ 1 : 40) against different *Salmonella* serotypes.

Serotype	Antibody type	Positive results (*N* = 400) *n* (%)
*S. *Typhi	Anti-TO	37 (9.25%)
*S. *Typhi	Anti-TH	41 (10.25%)
*S. *Paratyphi A	Anti-TH	2 (0.50%)
*S. *Paratyphi B	Anti-BH	15 (3.75%)

*N*: total number of participant blood donors; *n*: number of individuals with antibody titre ≥ 1 : 40 for respective serotype.

**Table 3:** Comparison of frequency and percentage of baseline antibody titres against different serotypes of *Salmonella enterica*.

Antigen	Total	Dilution end titres (*N* = 400)			
		1 : 40	1 : 80	1 : 160	1 : 320
*S. *Typhi “O”	37 (9.25%)	20 (5%)	14 (3.5%)	1 (0.25%)	2 (0.5%)
*S. *Typhi “H”	41 (10.25%)	11 (2.75%)	25 (6.25%)	5 (1.25%)	—
*S. *Paratyphi “AH”	2 (0.5%)	1 (0.25%)	1 (0.25%)	—	—
*S. *Paratyphi “BH”	15 (3.75%)	4 (1%)	7 (1.75%)	4 (1%)	—

*N*: total number of participant blood donors.
