# Supplementary material for: Baseline Antibody Titre against Salmonella enterica in Healthy Population of Mumbai, Maharashtra, India
Source: Int J Microbiol. 2017 Aug 31;2017:9042125. doi: 10.1155/2017/9042125 (PMC5610875; doi:10.1155/2017/9042125)
Supplement: Supplementary file 1 — The participant blood donors were surveyed using a questionnaire. Only those who had never been vaccinated with TAB or oral typhoid vaccine and who had not suffered from enteric fever in the past, were selected for the study. Participating blood donors were informed and explained about the study and consent was obtained. [file 9042125.f1.pdf]

**Survey Questionnaire**

Blood donors were surveyed using the following questionnaire:

**Donor details**

1) Name: 2) Donor ID:

3) Age: 4) Sex:

5) Date of Birth:

6) Address:

7) Occupation:

8) Telephone: Home: Work:

**General Health**

**In general, how would you describe your health?**

Excellent ( ) Very good ( ) Good ( ) Fair ( ) Poor ( )

**2) Any general complaints about your health lately?**

**Symptoms**

**1) Have you experienced a FEVER lately?**

Low grade ( ) High grade ( ) Intermittent ( ) Continuous ( ) Remittent ( )

If yes, mention the duration:

**2) On a scale of 1 to 10 (10 being the highest) describe if you have any of the following:**

Fatigue (   ) Anorexia (   ) Abdominal Pain (   ) Nausea/Vomiting (   ) Difficulty in passing stool (   )

Headache (   )

**3) Describe the following:**

a. Colour of stool:

b. Colour of urine:

c. Icterus:

d. Have you experienced weight loss over the past 2-3 months? (Quantify):

e. Tick if you have had any of these symptoms recently? :

| **Symptoms** | **Yes** | **No** |
|---|---|---|
| Fever | | |
| Abdominal pain | | |
| Constipation | | |
| Diarrhoea | | |
| Headache | | |
| Malaise | | |
| Coated tongue | | |

## Health Care History

**1. Any other CHRONIC condition you suffer from?**

Specify:

**2. Did you suffer from any chronic liver disease?**

**Yes (  ) No (  )**

**If yes, specify:**

**3. Do you suffer from any BLEEDING DISORDER that has required repeated BLOOD TRANSFUSION?**

Specify:

**4. Have you had any SURGERY in the past one year?**

Specify:

**5. Have you been tested for typhoid before?**

Yes (    )  No (    )

If yes specify the last date for test:

**6. Have you suffered from typhoid before?**

Yes (    )  No (    )

If yes, mention the year:

**7. Did you suffer from any of the following in the past one year?**

1. Malaria:    Yes (    ) No (    )

2. Hepatitis:    Yes (    ) No (    )

3. Brucellosis:  Yes (    ) No (    )

4. Tuberculosis: Yes (    ) No (    )

5: Dengue fever: Yes (    ) No (    )

**Personal History**

**1 Do you have any addictions?**

Alcohol (    ) Drugs (    ) Cigarettes (    ) Other (    )

**2 Have you ever used intra-venous drugs?**

Yes (    ) No (    )

**3. Have you ever been in contact with anyone infected with HIV, HCV or HBV?**

No (    ) Yes (    )

If yes, answer the following:

Mother (    ) Father (    ) Child (    ) Spouse (    ) Grandparents (    ) Others_________

**4. Needle-stick injury**

Yes (    ) No (    )

**5. Have you had any tattoos/piercings?**

Yes (    ) No (    )

**6. Have you ever received a blood transfusion or donated blood?**

Yes (    ) No (    )

If Yes, Specify the Date:

**7. Have you ever been in contact with any un-sterilized medical apparatus?**

Yes (    ) No (    )

If Yes, Specify:

Vaccination:

Unauthorized Abortion Centres

Other_______________

**8. Drug History**

1 Are you consuming any drug currently?

Yes (    ) No (    )

If Yes, Answer the following:

a. Name of Drug:

b. Dosage form:

c. Dose:

d. Time since consumption started:

**9. Vaccination history:**

Have you been vaccinated for typhoid before?

Yes ( ) No ( )

If yes,

1. Mention the type of vaccine:

TAB: ( ) Oral: ( ) Vi: ( )

2. Mention the year of vaccination:

**LABORATORY RESULTS:**

| **Test** | **Result: Agglutination** | **Titre (if agglutination present)** |
|---|---|---|
| ***Salmonella typhi* "O"** | Present/Absent | |
| ***Salmonella typhi* "H"** | Present/Absent | |
| ***Salmonella Paratyphi*"AH"** | Present/Absent | |
| ***Salmonella paratyphi*"BH"** | Present/Absent | |

.

**INFORMED CONSENT FORM:**

**Consent form for STS Project on Baseline antibody titre against *Salmonella enterica* in healthy population of Mumbai, Maharashtra, India. (Year 2014)**

**Name of the donor:………………………………**

**Sex:……… Date of Birth:…./…../……. Age…**

**Donor ID: ……………….……….**

I have been provided information regarding the study and the tests to be conducted on me and

have been explained about the implications of the test results. I have also been informed regarding the limitations and interpretation of results in a manner that I can understand.

I, hereby, give my consent to collect blood sample for the Widal test to be conducted on me in order to determine the results for the said study.

Signature/ Thumb impression: Date: _______________

________________________

(Name in capital letters)
